# Hippocampal PPARδ Overexpression or Activation Represses Stress-Induced Depressive Behaviors and Enhances Neurogenesis

**DOI:** 10.1093/ijnp/pyv083

**Published:** 2015-09-10

**Authors:** Miao-Jin Ji, Xu-Ben Yu, Zhen-Lin Mei, Yun-Qi An, Su-Su Tang, Mei Hu, Yan Long, Ming-Xing Miao, Qing-Hua Hu, Hong-Bin Sun, Ling-Yi Kong, Hao Hong

**Affiliations:** Department of Pharmacology (Ms Ji, Mr Yu, Ms Mei, Ms An, Ms Tang, Ms Hu, Dr Long, Dr Miao, Dr Hu, and Dr Hong), Jiangsu Key Laboratory of Drug Discovery for Metabolic Diseases (Ms Ji, Mr Yu, Ms Mei, Dr Hong, and Dr Sun), and State Key Laboratory of Natural Medicines (Ms Ji, Mr Yu, Ms Mei, Dr Kong, and Dr Hong), China Pharmaceutical University, Nanjing.

**Keywords:** Depression, hippocampal neurogenesis, peroxisome proliferator-activated receptors δ

## Abstract

**Background:**

Emerging data have demonstrated that peroxisome proliferator-activated receptor δ (PPARδ) activation confers a potentially neuroprotective role in some neurodegenerative diseases. However, whether PPARδ is involved in depression is unknown.

**Methods:**

In this study, PPARδ was firstly investigated in the chronic mild stress (CMS) and learned helplessness (LH) models of depression. The changes in depressive behaviors and hippocampal neurogenesis were investigated after PPARδ overexpression by microinfusion of the lentiviral vector, containing the coding sequence of mouse PPARδ (LV-PPARδ), into the bilateral dentate gyri of the hippocampus or PPARδ activation by repeated systemic administration of PPARδ agonist GW0742 (5 or 10mg/kg.d, i.p., for 21 d).

**Results:**

We found that both CMS and LH resulted in a significant decrease in the PPARδ expression in the hippocampi of mice, and this change was reversed by treatment with the antidepressant fluoxetine. PPARδ overexpression and PPARδ activation each suppressed the CMS- and LH-induced depressive-like behavior and produced an antidepressive effect. *In vivo* or *in vitro* studies also showed that both overexpression and activation of PPARδ enhanced proliferation or differentiation of neural stem cells in the hippocampi of mice.

**Conclusions:**

These results suggest that hippocampal PPARδ upregulation represses stress-induced depressive behaviors, accompanied by enhancement of neurogenesis.

## Introduction

Major depressive disorder is a multi-causal psychiatric disorder and affects up to 17% of the global population (Tanti and Belzung, 2010). Antidepressants are currently the main treatment for depression and depressive episodes. Antidepressants acting on the monoaminergic system are an effective way to treat depression and are developed based on the “monoaminergic hypothesis,” considering the deficiency of synaptic 5-hydroxytryptamine (5-HT, serotonin) or noradrenaline (NA) as the main cause of depression (Belmaker and Agam, 2008). Unfortunately, these antidepressants were ineffective for a substantial proportion of patients, while only 30% of patients were completely cured (Hamilton rating score ≤7; Wong and Licinio, 2001; Shelton et al., 2010). The incomplete understanding of the depressive pathophysiology has limited the development of highly effective drugs against this disorder. Thus, developing novel pharmacological targets, beyond the serotoninergic and noradrenergic systems, is presently considered a promising strategy to treat depression and related mood disorders (Leggio et al., 2013).

Peroxisome proliferator-activated receptors δ (PPARδ, also called PPARβ), which is also known as nuclear receptor 1C2 (NR1C2), is one of three PPARs (the others are PPARα and PPARγ). They are the part of the nuclear receptor superfamily of transcription factors. After activation, PPARs undergo heterodimerisation with retinoid X receptors and regulate the expression pattern of the target genes containing peroxisome proliferator-responsive elements. PPARδ is a critical regulator of diverse processes, including lipid and glucose homeostasis, inflammation, and cell proliferation and differentiation (Feige et al., 2006; Straus and Glass, 2007). Amazingly, in addition to its biological activity in peripheral organs, the PPARδ is also highly expressed in the brain (Braissant et al., 1996) and its deletion in mice is associated with the alteration in myelination of the corpus callosum (Peters et al., 2000). To date, the neuroprotective benefits of PPARδ agonists have been reported in experimental models of stroke (Arsenijevic et al., 2006; Pialat, et al., 2007), Alzheimer’s disease (Kalinin et al., 2009), Parkinson’s disease (Martin et al., 2013; Das et al., 2014), autoimmune encephalomyelitis (Kanakasabai et al., 2011), and spinal cord injury (Paterniti et al., 2010; Tsai et al., 2014). However, no reports on the association of PPARδ with depression have been found yet. Here we investigated the effect of hippocampal PPARδ overexpression or stimulation on depressive behaviors and hippocampal neurogenesis.

## Materials and Methods

### Animals and Reagents

Male Institute of Cancer Research (ICR) mice (Yangzhou University Medical Center, China), weighing 18–22g (6–8 weeks), were housed with maintained temperature (22±2°C), humidity (55±5%), and a 12h light/dark cycle. Food and water were freely available unless otherwise noted. All procedures were performed according to the *National Institutes of Health Guide for the Care and Use of Laboratory Animals* (Laevis and Tropicials, 1996) and approved by the Animal Care and Use Committee of China Pharmaceutical University. [4-[[[2-[3-Fluoro-4-(trifluoromethyl)phenyl]-4-methyl-5-thiazolyl]methyl]thio]-2-methylphenoxy]acetic acid (GW0742) (Santa Cruz Biotechnology) was dissolved in 10% 4,6-diamidino-2-phenylindole (DMSO) (Sinopharm Chemical Reagent) saline solution (Vehicle, Veh), while bromodeoxyuridine (BrdU; Sigma-Aldrich) and fluoxetine hydrochloride (Changzhou Siyao Pharmaceuticals) were prepared in saline. Other reagents have been described in the methods.

### Mouse Surgery and Lentivirus Microinfusion

Mice were anesthetized with trichloroacetaldehyde hydrate (350mg/kg, i.p.) and placed in a stereotaxic device. A 30 gauge infusion cannula was inserted into the dentate gyrus (DG; anteroposterior, -2.3mm; medial-lateral, ±1.3mm; dorsal-ventral, -2.0mm; Munoz et al., 2005) on each side. Lentiviral vectors (2×10^9^ TU/μl, 2 μl/side) containing PPARδ plus enhanced green fluorescent protein (EGFP) or EGFP alone were infused (0.2 μl/min) using a micro-injection pump (CMA402 Suringo Pump, Dakumar Machinery, Sweden). Injectors were left intact for 5min in place after completing the injection to ensure diffusion from the syringe tip. Behavioral tests and immunohistochemistry or immunofluorescence assays were performed on the scheduled time after the infusion (Figure 2A).

### Chronic Mild Stress and Learned Helplessness

Chronic mild stress (CMS) mice were subjected to various mild stressors, including food and/or water deprivation, wet bedding, reversal of the day/night light cycle, forced swimming, restraint, and stroboscopic illumination for a period ranging from 10min to 24h in a schedule of 3 weeks based on published studies (Ducottet et al., 2003). The program was repeated thereafter from week 1. The CMS exposure mice were individually housed, while control mice were group housed and placed in the palm of the hand daily for 30 s.

During the 3-day training, learned helplessness (LH) mice were exposed to 360 inescapable footshocks (0.3 mA, 4-s duration, at an interval of 6 s) over a 1-h session (Caldarone et al., 2000). Another group was treated in a similar manner but without the electrical shock. A test consisting of 30 trials with an interval (30 s) was conducted 24h after the last training. Each trial (24 s duration) consisted of 2 s of voice stimulus, following a 4 s footshock. The Gemini Avoidance System was used to record the number of escape failures (i.e. failed to escape from the footshock) and escape latencies (i.e. latency to escape after footshock onset). More than 20 failures were considered as LH. On the tenth day, mice were subjected to a reminder session, which consisted of 10 inescapable shocks (0.3 mA, 4s), while the control mice received similar treatment without any shocks.

### Neural Stem Cells (NSCs) Proliferation Assay

NSCs (1×10^4^ cells/well) grown on 96-well plates with 100 μl proliferation medium were cultured in neurosphere for 72h, and then the cells were incubated with the indicated regents for another 72h. NSCs proliferation was assessed by 3-(4, 5-dimethythiazole-2-yl)-2, 5-diphenyl-tetrazolium bromide (MTT) and cell counting kit (CCK-8, Beyotime Biotechnology) assays according to the manufacturers’ protocols. The data indicating cell proliferation was expressed as a percentage compared with control. We also observed BrdU incorporation for the monolayer-cultured NSCs. Briefly, NSCs were cultured on polyornithine/laminin-coated 24-well culture plates and cultured as monolayers for 72h. To label the dividing cells, 10μM BrdU (Sigma Aldrich) was added into medium during the last 4h of culture. NSCs were then fixed in 4% paraformaldehyde (diluted with PBS) for 30min and BrdU^+^ cells were counted according to a previous description (Guo et al., 2011).

### NSCs Differentiation

NSCs were plated on polyornithine/laminin-coated 24-well plates with growth factor-free dulbecco’s modified eagle medium (DMEM)/F12 (1:1; Hyclone) medium containing 0.5% fetal bovine serum and 2% B27. On the fifth day after treatment, cells were fixed in 4% paraformaldehyde and incubated with neuronal marker neuronal nuclear antigen (NeuN) or astrocytic marker glial fibrillary acidic protein (GFAP) antibodies. The images were acquired using a fluorescence microscope (Olympus DP72). The number of NeuN^+^ or GFAP^+^ cells was counted using a 20×objective and fluorescence filters in four selected fields, systematically placed in the same positions relative to the edges of the 24-well culture plate. 4’,6-Diamidino-2-phenylindole (DAPI^+^) cells were used to count the total number of cells. The image was analyzed with Image-Pro Plus software (Media Cybernetics). The proportion of cells positive for specific markers was calculated to the total number.

### Immunohistochemistry/Immunofluorescence Analysis

For analyzing hippocampal neurogenesis and neural differentiation, mice were administered four injections of BrdU (50mg/kg, i.p., every 2h) on the last day of CMS exposure. Animals were euthanized with chloral hydrate (30mg/kg, i.p.) 15 d after the last BrdU administration and were transcardially perfused (0.1M PBS followed by 4% paraformaldehyde). The brains were postfixed in 4% paraformaldehyde overnight, and dehydrated with 30% sucrose over 2 days. Serial sections (35 μm) were cut throughout the hippocampi using an oscillating tissue slicer and preserved in normal saline. Then the sections were incubated with rat polyclonal antibody anti-BrdU (1:40, Abcam), rabbit polyclonal antibody anti-NeuN (1:400, Chemicon), and rabbit polyclonal antibody anti-GFAP (1:100, Chemicon) under 4℃ overnight. We used the following secondary antibodies: cyanin 3 (1:500, Beyotime Biotechnology), Alexa Fluor 350 (1:500, Beyotime Biotechnology), DyLight 405 (1:100, Bioworld Biotechnology), and Alexa Fluor 647(1:500, Beyotime Biotechnology). Fluorescent signals were detected using a fluorescence microscope (Olympus DP72). Cells were counted by another experimenter unaware of the treatment group as described previously (Jeong-Sik et al., 2009). Briefly, every ninth section was kept for BrdU immunohistochemistry. According to the Franklin and Paxinos atlas of the mouse brain, the dorsal hippocampus was determined as AP: -0.94 to -2.30 and the ventral hippocampus was determined as AP: -2.46 to -3.80. Cells in the DG and hilus were counted through a 40× objective lens in each section and multiplied by ten, regarded as the total quantity of labeled cells. For the *in vitro* experiment, the primary antibodies used were as follows: rabbit anti-NeuN (1:200, Chemicon), rabbit anti-GFAP (1:100, Chemicon), and rat anti-nestin (1:100, Chemicon). The number of marker-positive cells as well as total cells (DAPI^+^) or EGFP-infected cells (EGFP^+^) was quantified using a fluorescence microscope. The quantification was carried out using Image-Pro Plus software. The percentage of differentiated cells was calculated as the number of marker-positive cells divided by the total cells.

### Statistical Analysis

Data shown are expressed as mean ± standard error of the mean. The data normality was assessed by the Kolmogorov-Smirnov test in SPSS. Statistical analyses among multiple groups were performed with one-way ANOVA followed by Scheffe’s post hoc test. The physical state index data between two groups was compared by the Wilcoxon–Mann–Whitney nonparametric test. Others comparisons were made with the two-tailed *t*-test. Data were considered significant at *p* < 0.05.

## Results

### Hippocampal PPARδExpression is Downregulated in Mice Exposed to CMS or LH

**Figure 1.**
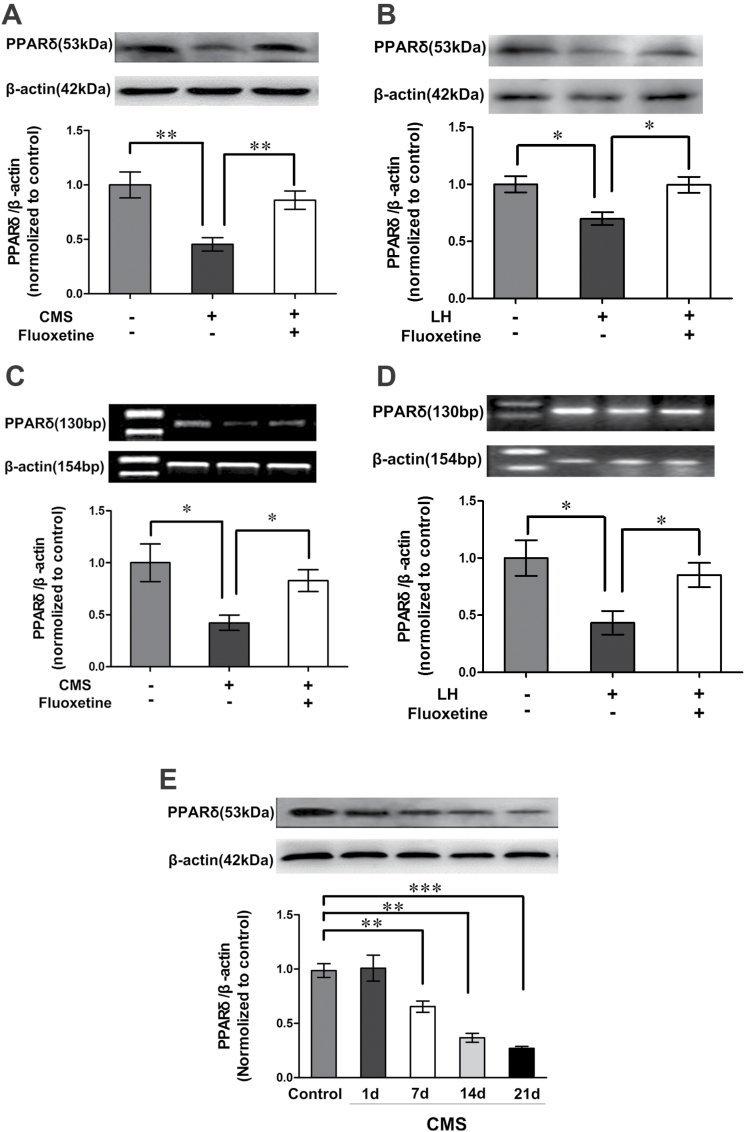
Hippocampal peroxisome proliferator-activated receptor δ (PPARδ) expression is downregulated in mice exposed to chronic mild stress (CMS) or learned helplessness (LH). Immunoblots showing PPARδ protein level in the hippocampus of (A) CMS- or (B) LH-induced depression mice (n = 4). Reverse transcription-polymerase chain reaction analysis showing PPARδ mRNA in the hippocampi of (C) CMS or (D) LH depressed mice (n = 3–4). (E) Immunoblots showing hippocampal PPARδ protein of the mice treated with CMS for 1, 7, 14, or 21 d (n = 4). Data are mean ± standard error of the mean; (A, C) ^*^ *p* < 0.05, ^**^ *p* < 0.01, compared with CMS; (B, D) ^*^ *p* < 0.05, ^**^ *p* < 0.01, compared with LH; (E) ^**^ *p* < 0.01, ^***^ *p* < 0.001, compared with control.

Stressful stimuli play a causal role in depression, and CMS have long been used to induce depressive-like behaviors in mice (Hammen, 2005; Bartolomucci and Leopardi, 2009). Accordingly, we investigated whether CMS changes hippocampal PPARδ expression in mice. Surprisingly, mice exposed to CMS for 6 weeks displayed a significant decrease in hippocampal PPARδ levels relative to control mice [*F*_*(2,9*)_ = 9.593; *p* = 0.0066, Figure 1A]. The decrease in hippocampal PPARδ was also observed in the mice exposed to the stress of LH [*F*_*(2,9*)_ = 6.789; *p* = 0.0159, Figure 1B]. Furthermore, chronic administration of ﬂuoxetine (10 mg·kg^-1^·d^-1^, i.p.) for 3 weeks reversed the CMS- or LH-induced changes in hippocampal PPARδ, as seen by Western blot [CMS, *F*_*(2,9*)_ = 9.593, *p* = 0.0080; LH, *F*_*(2,9*)_ = 6.789, *p* = 0.0165; Figure 1A and B]. To confirm these findings, we performed a reverse transcription-polymerase chain reaction (RT-PCR) assay. The PPARδ mRNA level was consistent with its protein level in the hippocampi of mice exposed to CMS or LH (CMS, r = 0.997, *p* < 0.001; LH, r = 0.672, *p* = 0.048; Figure 1C and D). To further confirm the effect of stress on hippocampal PPARδ, we exposed adult mice to CMS for 1, 7, 14, or 21 d, and found that hippocampal PPARδ displayed gradual decreases over the CMS exposure time [*F*_*(4,15*)_ = 25.32; control vs 1 d, *p* = 0.8721; control vs 7 d, *p* = 0.0065; control vs 14 d, *p* = 0.0020; control vs 21 d, *p* < 0.001; 1 d vs 7 d, *p* = 0.0345; 7 d vs 14 d, *p* = 0.0048; 14 d vs 21 d, *p* = 0.0735; Figure 1E]. Taken together, these findings strongly suggest that PPARδ is affected by chronic stress exposure.

### Hippocampal PPARδOverexpression or Activation Decreases Depressive Behaviors

**Figure 2.**
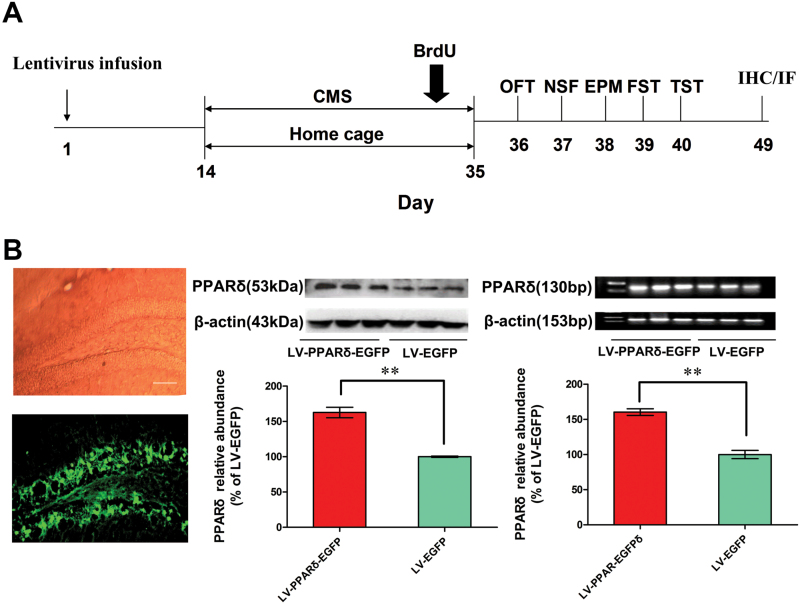

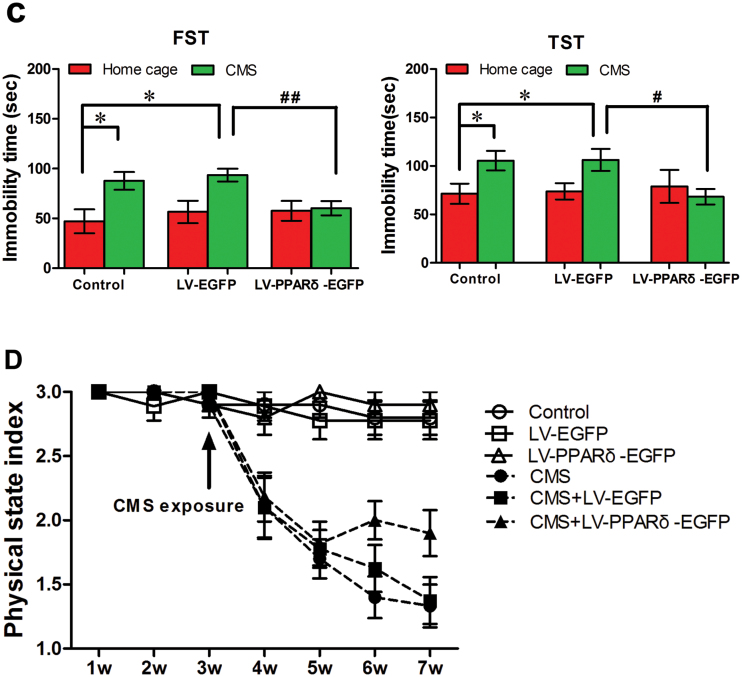

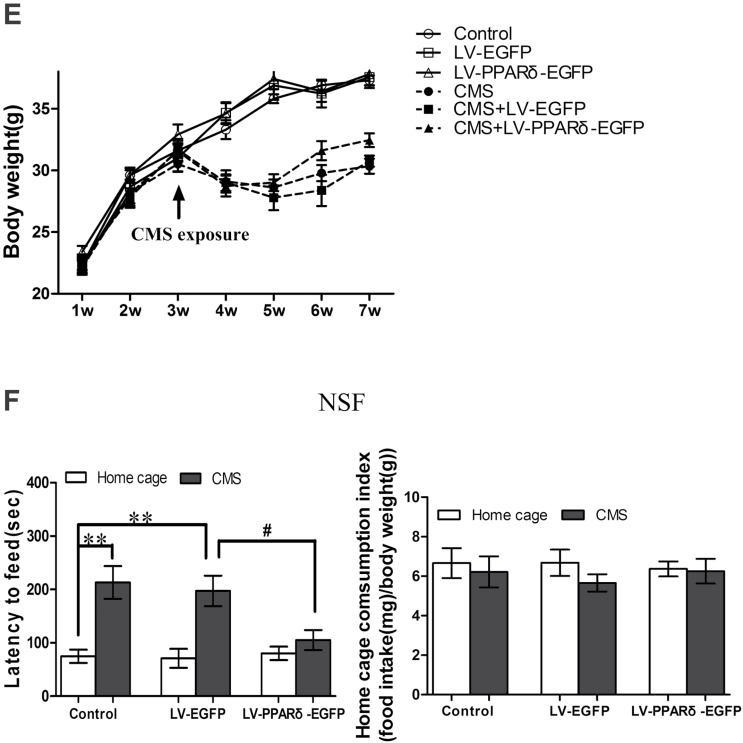

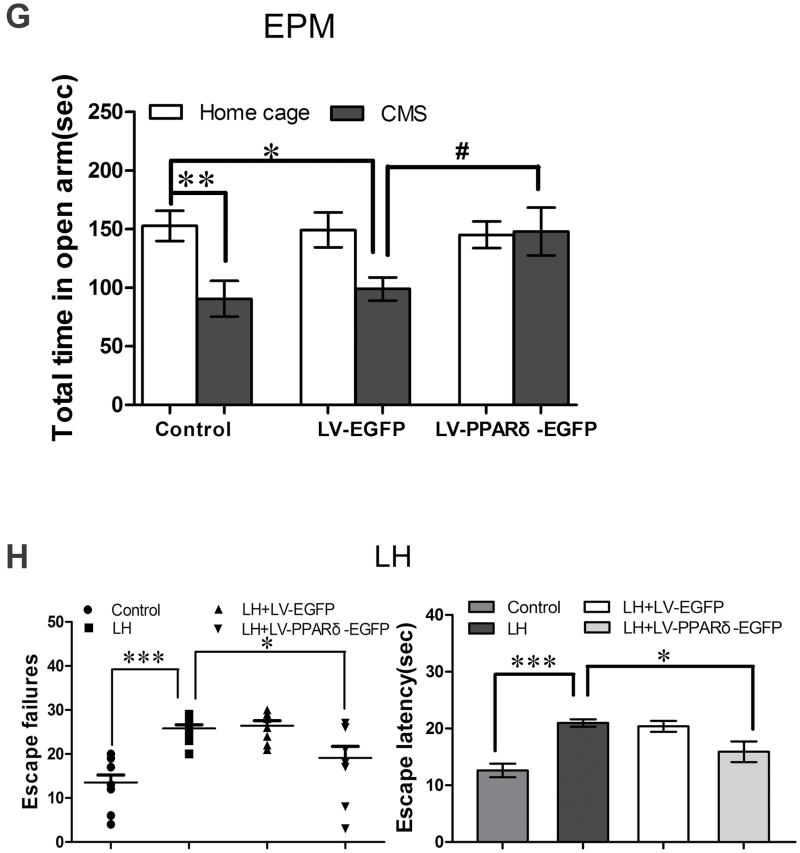
Hippocampal peroxisome proliferator-activated receptor δ (PPARδ) overexpression decreases depressive behaviors. (A) Schematic timeline of the experimental procedure. OFT, open field test; NSF, novelty-suppressed feeding; EPM, elevated plus maze; FST, forced swimming test; TST, tail suspension test; IHC, immunohistochemistry; IF, immunofluorescence. (B) Shown are representative dentate gyrus (DG) area with transfection of a lentiviral vector that selectively expresses PPARδ with enhanced green fluorescent protein (LV-PPARδ-EGFP), PPARδ protein, or mRNA in the hippocampus (n = 3). Shown are the (C) immobility time in the forced swim test (FST) and tail suspension test (TST), (D) physical state index, (E) body weight, (F) latency to feed and home cage consumption index in NSF test, and (G) total time spent in open arms in EPM in the mice intrahippocampally microinjected with LV-PPARδ-EGFP (2×10^9^ TU, 2μl/side), followed 2 weeks later by chronic mild stress (CMS) for 21 d (n = 8–10). (H) Shown are escape failures and escape latency in the learned helplessness (LH) test (n = 8–10). Data are mean±standard error of the mean. (B) ^**^ *p* < 0.01 compared with a lentiviral vector expressing EGFP alone (LV-EGFP); (C-G)^*^ *p* < 0.05, ^**^ *p* < 0.01 compared with control; ^*#*^ *p* < 0.05, ^*##*^ *p* < 0.01, compared with CMS plus LV-EGFP; (H) ^*^ *p* < 0.05, ^**^ *p* < 0.01 compared with LH.

We generated a lentiviral vector that selectively expresses PPARδ with EGFP and named it LV-PPARδ-EGFP. As a control, we also generated a lentiviral vector expressing EGFP alone (LV-EGFP). LV-PPARδ-EGFP or LV-EGFP was infused into the bilateral DG of mice by stereotaxic injection (Figure 2A). On the fifth day after transfection, numerous EGFP positive cells and increased PPARδ protein and mRNA were observed in the DG (*p* = 0.001; Figure 2B). After 2 weeks, we treated the mice with CMS for 21 days and tested their behaviors. Mice treated with CMS + LV-PPARδ-EGFP displayed a significantly decreased immobility time in the forced swimming test [*F*_*(5,48*)_ = 3.577, *p* = 0.0038; Figure 2C] and tail suspension test [*F*_*(5,48*)_ = 2.096, *p* = 0.0157; Figure 2C], relative to those with CMS + LV-EGFP. Hippocampal PPARδ levels in mice overexpressing PPARδ were significantly decreased after CMS exposure [*F*_*(5,18*)_ = 8.617, *p* = 0.0234], but were still significant higher than those of age-matched CMS-exposed mice [*F*_*(5,18*)_ = 8.617, *p* = 0.0148]. PPARδ overexpression improved the physical state index [week 7, *F*_*(5,50*)_ = 24.22, *p* = 0.0228; Figure 2D] and weight gain [week 7, *F*_*(5,49*)_ = 25.76, *p* = 0.0345; Figure 2E] in stressed groups as well. Novelty-suppressed feeding (NSF) and elevated plus maze (EPM) tests were performed to observe the effect of PPARδ overexpression on anxiety-like behavior in mice exposed to CMS. The latency to feed was significantly reduced in the CMS + LV-PPARδ-EGFP mice compared to the CMS + LV-EGFP mice [*F*_*(5,48*)_ = 9.595, *p* = 0.0179; Figure 2F], but the home cage consumption index didn’t change [*F*_*(5,48*)_ = 0.3306, *p* = 0.4457; Figure 2F] and the time spent in the open arm of EPM remarkably increased in the CMS + LV-PPARδ-EGFP mice [*F*_*(5,45*)_ = 3.838, *p* = 0.0481; Figure 2G]. No effect of PPARδ overexpression on the locomotor activity was observed [line crossing: F_(5,48)_ = 0.4612; control vs LV-PPARδ-EGFP, *p* = 0.2565; CMS vs CMS + LV-PPARδ-EGFP, *p* = 0.8204; CMS + LV-EGFP vs CMS + LV-PPARδ-EGFP, *p* = 0.4311; total distance: F_(5,48)_ = 0.2919; control vs LV-PPARδ-EGFP, *p* = 0.3236; CMS vs CMS + LV-PPARδ-EGFP, *p* = 0.6457; CMS + LV-EGFP vs CMS + LV-PPARδ-EGFP, *p* = 0.7446]. Moreover, LH animals receiving LV-PPARδ-EGFP showed significant decreases in failure number and escape latency relative to LH-only mice [failure numbers, *F*_*(3,35*)_ = 12.32, *p* = 0.0236; escape latency, *F*_*(3,35*)_ = 10.14, *p* = 0.0485; Figure 2H], but not to that of the control animals [failure numbers, *F*_*(3,35*)_ = 12.32, *p* = 0.2307; escape latency, *F*_*(3,35*)_ = 10.14, *p* = 0.2439; Figure 2H], which further identifies an antidepressive effect of hippocampal PPARδ overexpression.

To further verify the role of PPARδ in regulating depressive behaviors, we investigated the antidepressive effect of GW0742, a selective PPARδ agonist, in mice exposed to CMS. PPARδ agonist GW0742 treatment (5 or 10mg/kg, i.p.) completely blocked CMS-induced immobility time prolongation in the tail suspension test [*F*_*(3,30*)_ = 5.180; CMS + 5mg/kg GW0742 vs CMS + Veh, *p* = 0.0333; CMS + 10mg/kg GW0742 vs CMS + Veh, *p* = 0.0161; CMS + 5mg/kg GW0742 vs control, *p* = 0.4703; CMS + 10mg/kg GW0742 vs control, *p* = 0.6777; Supplementary Figure S1A] and body weight loss [*F*_*(3,33*)_ = 5.974; control vs CMS + Veh, *p* = 0.0032; CMS + 5mg/kg GW0742 vs CMS + Veh, *p* = 0.1923; CMS + 10mg/kg GW0742 vs CMS + Veh, *p* = 0.0015; CMS + 5mg/kg GW0742 vs control, *p* = 0.5235; CMS + 10mg/kg GW0742 vs control, *p* = 0.7103]. In the NSF test, GW0742 treatment markedly reduced the latency to feed compared with CMS mice [*F*_*(3,28*)_ = 4.986; CMS + 5mg/kg GW0742 vs CMS + Veh, *p* = 0.0197; CMS + 10mg/kg GW0742 vs CMS + Veh, *p* = 0.0125; CMS + 5mg/kg GW0742 vs control, *p* = 0.2856; CMS + 10mg/kg GW0742 vs control, *p* = 0.5609; Supplementary Figure S1B], without changing mice home cage consumption index [*F*_*(3,28*)_ = 0.03891; CMS + 5mg/kg GW0742 vs CMS + Veh, *p* = 0.9641; CMS + 10mg/kg GW0742 vs CMS + Veh, *p* = 0.7583; Supplementary Figure S1B]. GW0742 treatment also significantly reduced LH-induced depressive behaviors in mice, and no effect of GW0742 treatment on locomotor activity was observed in mice [line crossing: F_(3,28)_ = 0.3206; CMS vs CMS + 5mg/kg GW0742, *p* = 0.4456; CMS vs CMS + 10mg/kg GW0742, *p* = 0.4941; total distance: F_(5,48)_ = 0.1601; CMS vs CMS + 5mg/kg GW0742, *p* = 0.6640; CMS vs CMS + 10mg/kg GW0742, *p* = 0.9840].

### PPARδOverexpression or Activation Enhances Hippocampal Neurogenesis

**Figure 3.**
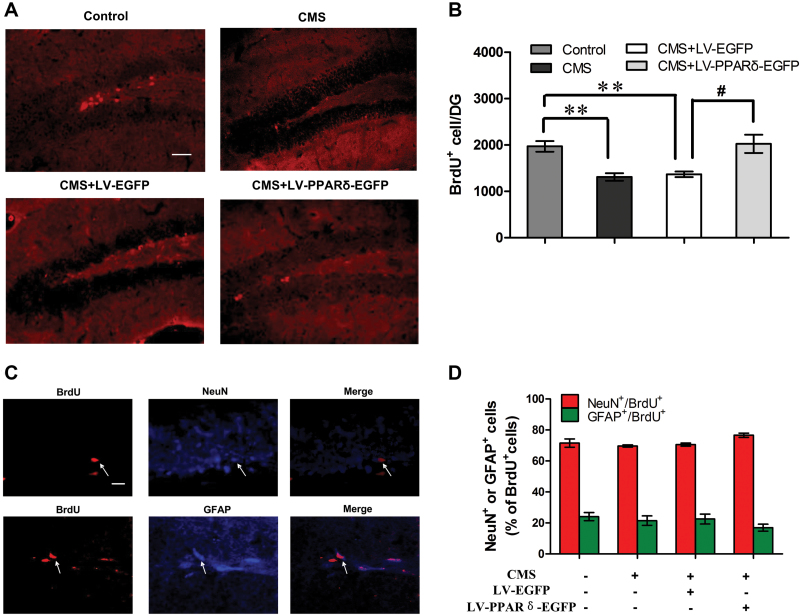
Peroxisome proliferator-activated receptor δ (PPARδ) overexpression enhances hippocampal neurogenesis. (A) Representative cells and(B) quantification of bromodeoxyuridine (BrdU)-labeled cells (red) in the dentate gyrus (DG) from control or chronic mild stress (CMS) mice with a lentiviral vector expressing enhanced green fluorescent protein alone (LV-EGFP) or a lentiviral vector that selectively expresses PPARδ with EGFP (LV-PPARδ-EGFP; n = 4). Scale bars = 50 μm. (C) Phenotype of BrdU-positive cells in the DG of CMS + LV-PPARδ-EGFP mice, and higher magnification of cells double-labeled for BrdU (red; left) and the neuronal marker nuclear antigen (NeuN; blue, middle and top) or the glialmarker glial fibrillary acidic protein (GFAP; blue, middle and bottom). Scale bars = 20 μm. (D) Percentages of neuronal and glial cells labeled by BrdU in the DG of control or CMS mice treated with LV-EGFP or LV-PPARδ-EGFP (n = 4). Data are mean ± standard error of the mean. ^*^ *p < 0.01*, compared with control, ^#^ *p* < 0.05, compared with CMS plus LV-EGFP.

Because of the putative involvement of hippocampal neurogenesis in the control of anxiety and depressive-like states, we next assessed the effect of PPARδ overexpression or activation on neurogenesis in CMS mice using BrdU labeling analysis. As shown in Figure 3A and B, the number of BrdU^+^ cells in the DG in CMS or CMS + LV-EGFP mice significantly decreased relative to control mice, whereas the number of BrdU^+^ cells in the DG in the mice treated with CMS + LV-PPARδ-EGFP was increased by 48.0% compared to the mice treated with CMS + LV-EGFP [*F*_*(3,12*)_ = 9.403, *p* = 0.0150]. We also assessed BrdU^+^ cells in the dorsal and ventral DG of the hippocampus. Microinjection of LV-PPARδ-EGFP could reverse CMS-induced BrdU^+^ cells’ decline both in the dorsal and ventral DG of the hipppocampus [dorsal hippocampus: F_(3,12)_ = 14.86; control vs CMS, *p* = 0.0038; CMS vs CMS + LV-EGFP, *p* = 0.8576; CMS vs CMS + LV-PPARδ-EGFP, *p* = 0.0010; ventral hippocampus: F_(3,12)_ = 22.10; control vs CMS, *p <* 0.001; CMS vs CMS + LV-EGFP, *p* = 0.7290; CMS vs CMS + LV-PPARδ-EGFP, *p* = 0.0026]. The phenotype of BrdU+ cells was determined by double labeling of BrdU and NeuN or GFAP. No significant differences in the percentage of NeuN^+^/BrdU^+^ (*p* = 0.2603) or GFAP^+^/BrdU^+^ (*p* = 0.2060) were observed among the various groups [NeuN^+^/BrdU^+^, *F*_*(3,12*)_ = 1.377; GFAP^+^/BrdU^+^, *F*_*(3,12*)_ = 1.182; Figure 3C and D]. In addition, GW0742 treatment significantly blocked the CMS-induced reduction in the number of BrdU^+^ cells in the DG [*F*_*(3,12*)_ = 4.556; CMS + 5mg/kg GW0742 vs CMS + Veh, *p* = 0.0310; CMS + 10mg/kg GW0742 vs CMS + Veh, *p* = 0.0475; dorsal hippocampus: F_(3,12)_ = 4.706; CMS + Veh vs CMS + 5mg/kg GW0742, *p* = 0.3560; CMS + Veh vs CMS + 10mg/kg GW0742, *p* = 0.0491; ventral hippocampus: CMS + Veh vs CMS + 5mg/kg GW0742, *p* = 0.0140; CMS + Veh vs CMS + 10mg/kg GW0742, *p* = 0.0404; Supplementary Figure S2A and B].

### PPARδOverexpression or Activation Promoted Proliferation and Differentiation of Neural Stem Cells *In Vitro*

**Figure 4.**
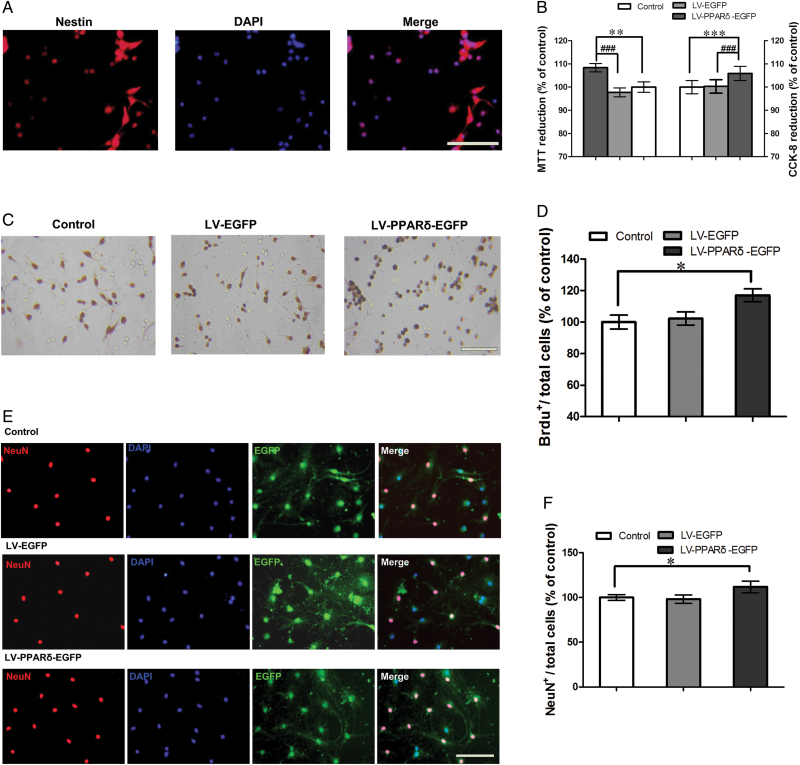
Peroxisome proliferator-activated receptor δ (PPARδ) overexpression promoted proliferation and differentiation of neural stem cells (NSCs) *in vitro*. (A) Shown is the protein marker nestin, expressed by NSCs. (B) The coding sequence of mouse PPARδ (LV-PPARδ) increases proliferation determined by 3-(4, 5-dimethythiazole-2-yl)-2, 5-diphenyl-tetrazolium bromide (MTT) and cell counting kit (CCK-8) assays (n = 6). (C) Representatives of bromodeoxyuridine positive (BrdU^+^)-labeled cells of the NSCs after treatment with a lentiviral vector that selectively expresses PPARδ with enhanced green fluorescent protein (LV-PPARδ-EGFP). (D) Statistical graph shows the number of BrdU^+^ cells in different groups (n = 6). (E) Immunocytochemistry for neuronal marker neuronal nuclear antigen (NeuN) was used to assess neural differentiation in NSCs treated with LV-PPARδ. (F) Shown are statistical data of NeuN^+^-positive neurons in different groups (n = 6). Data are mean ± standard error of the mean. ^*^ *p < 0.05,* ^**^ *p < 0.01,* ^***^ *p <* 0.001, compared with control; ^*#*^ *p <* 0.001, compared with a lentiviral vector expressing EGFP alone (LV-EGFP). Scale bars = 50 μm.

To further support the *in vivo* results, we observed the effect of PPARδ overexpression or stimulation on the proliferation and differentiation of neural stem cells *in vitro*. In a floating culture medium, NSCs from mice hippocampi showed neurosphere formation and expressed nestin (Figure 4A). CCK-8 and MTT reduction assays showed that cell proliferation was pronouncedly increased in the NSCs transfected with LV-PPARδ-EGFP relative to controls [MTT: *F*_*(2,15*)_ = 7.798; control vs LV-EGFP, *p =* 0.6108; control vs LV-PPARδ-EGFP, *p* = 0.003; LV-EGFP vs LV-PPARδ-EGFP, *p <* 0.001; CCK-8: *F*_*(2,15*)_ = 12.669; control vs LV-EGFP, *p =* 0.9476; control vs LV-PPARδ-EGFP, *p <* 0.001; LV-EGFP vs LV-PPARδ-EGFP, *p <* 0.001; Figure 4B]. Consistent with LV-PPARδ-EGFP, GW0742 significantly increased cell proliferation [MTT: *F*_*(2,15*)_ = 6.192; GW0742 0.1μM vs control, 1.14±0.13 fold, *p* = 0.0041; GW0742 10 μM vs control, 1.15±0.12 fold, *p* = 0.0023; CCK-8: *F*_*(2,15*)_ = 6.339; GW0742 0.1 μM vs control: 1.04±0.02 fold，*p* = 0.0062; GW0742 10 μM vs control, 1.05±0.04 fold，*p* = 0.0099). Similarly, the BrdU incorporation experiment showed increased BrdU^+^ cells in monolayer-cultured NSCs [LV-PPARδ-EGFP: *F*_*(2,15*)_ = 4.833; control vs LV-EGFP, *p* = 0.7123; control vs LV-PPARδ-EGFP, *p =* 0.0103; Figure 4C and D; GW0742: *F*_*(2,15*)_ = 5.941; control vs GW0742 0.1 μM, *p* = 0.0259; control vs GW0742 10 μM, *p* = 0.0101; Supplementary Figure S3A and B).

We also investigated the effect of PPARδ overexpression or stimulation on cell differentiation in cultured NSCs. Results showed PPARδ overexpression significantly increased the percentage of NeuN^+^/total cells [*F*_*(2,15*)_ = 4.316; control vs LV-EGFP, *p* = 0.758; control vs LV-PPARδ-EGFP, *p* = 0.0105; Figure 4E and F]. Similarly, GW0742 treatment (0.1 or 10 μM) substantially increased the percentage of NeuN^+^/total cells [*F*_*(2,15*)_ = 6.193; control vs GW0742 0.1 μM, *p* = 0.0085; control v*s* GW0742 10 μM, *p* = 0.0072; Supplementary Figure S3C and D]. Neither PPARδ overexpression nor activation changed the percentage of GFAP^+^/total cells [LV-PPARδ-EGFP：*F*_*(2，15*)_ = 0.01780;control vs LV-EGFP, 0.99±0.11 fold，*p* = 0.8363; control vs LV-PPARδ-EGFP, 0.92±0.11, *p* = 0.1546; GW0742: *F*_*(2,15*)_ = 0.3737; control vs GW0742 0.1 μM, 0.91±0.10 fold，*p* = 0.0789; control vs GW0742 10 μM, 0.92±0.16 fold，*p* = 0.2292). These results indicate that PPARδ overexpression or stimulation promotes neuronal differentiation *in vitro*.

## Discussion

The major finding of our study is that chronic stress, such as CMS and LH, led to significant reductions of PPARδ protein and mRNA in the adult hippocampus. Both overexpression and activation of PPARδ inhibited CMS- or LH-induced depressive-like behaviors, showing an antidepressive effect, and the two treatment regimens also enhanced proliferation or differentiation of NSCs in the hippocampus, suggesting an antidepressive effect of PPARδ is accompanied by increases in neurogenesis.

Accumulating studies have supported a strong association between stress and depression, and stress paradigms have long been used to model the depressive disorder (Martinowich et al., 2007; Bartolomucci and Leopardi, 2009). Intriguingly, two kinds of classic stressful stimuli, CMS and LH, both led to significant decreases of PPARδ mRNA and protein, whereas the antidepressant ﬂuoxetine reversed such alterations. These evidences strongly suggest that PPARδ might be involved in depression occurrences. PPARδ is expressed in all the major cell types within the CNS, including astrocytes, endothelial cells, microglia, neurons, and oligodendrocytes. PPARδ mRNA and protein are expressed throughout the brain, with particularly high levels in the hippocampus, entorhinal cortex, and hypothalamus, but lower levels in the corpus callosum and caudate putamen (Woods et al., 2003; Hiqashiyama et al., 2007). PPARδ shows a relatively high neuronal expression compared with the other PPAR subtypes, such as PPARα and PPARγ (Lemberger et al., 1996). Herein, we found that LV-PPARδ delivered into the hippocampal DG upregulated PPARδ and produced a significant antidepressive-like effect in separate behavioral paradigms. A similar effect was mimicked by repeated treatment with the selective PPARδ agonist GW0742, which can penetrate the brain blood barrier (Sznaidman et al., 2003). In addition, because of the frequent intermixture of symptoms of depression and anxiety in human beings, depression often co-occurred with anxiety in clinical conditions, so we observed the animal performance in the NSF test, which uses the conflict between the fear in a new field and the drive to eat to test the animal’s anxiety state. Anxiety-like behavior was significantly alleviated in the depressive-like mice treated with LV-PPARδ or GW0742. Collectively, PPARδ upregulation showed a potential antidepressant effect.

Adult hippocampal neurogenesis, the formation of new neurons in the DG of the adult brain, can be regulated by stress and antidepressant treatment, and has consistently been implicated in the behavioral neurobiology of stress-related disorders, especially depression and anxiety (Gould et al., 1999; Malberg et al., 2000). Humans with depression have decreased hippocampal neurogenesis that results in hippocampal atrophy (Small et al., 2011; Fotuhi et al., 2012). As with the delayed effect of antidepressants in clinical application, antidepressants promoting hippocampal neurogenesis also have a lag time in experimental animals (Malberg et al., 2000), reflecting the time of neuronal maturation in the adult hippocampus (Ming and Song, 2011). Although accumulating studies suggest that neurogenesis deficiency is involved in depressive behaviors, their causal relationship remains controversial, since several studies have shown that eliminating neurogenesis has no effect on behavioral features (David et al., 2009; Surget et al., 2011; Groves et al., 2013; Jednyak et al., 2014). Interestingly, these conflicting results can be elucidated by the hypothesis that the extent of neurogenesis related to an antidepressant might depend on the degree of stress system alterations for different subjects (O’Leary and Cryan, 2014). Recent studies have shown that neurogenesis changes specific to a dorsal/ventral subregion are associated with observed behavioral phenotypes. It has been reported the dorsal hippocampus is associated with cognitive functions, while the ventral hippocampus relates to stress, emotion, etc. (Fanselow and Dong, 2010; O’Leary and Cryan, 2014). PPARδ activation has also been shown to enhance hippocampal-based learning and memory in mice (Kobilo et al., 2011; Yu et al., 2014). This effect might be associated with hippocampal dorsal neurogenesis, regulated by PPARδ. It has been reported that the activation of PPARδ induces oligodendrocyte differentiation and enhances neuronal maturation in cultured cell models of neurogenesis (Saluja et al., 2001; D’Angelo et al., 2011). In addition, the retinoic acid–activated PPARδ pathway is critical for promoting differentiation of neuronal progenitor cells to mature neurons (Cimini and Cerù, 2008; Kobilo et al., 2011; Yu et al., 2012). Here, our *in vivo* study showed that PPARδ overexpression or activation pronouncedly promoted the proliferation of hippocampal stem cells, and induced both neuonal and glial differentiation, whereas the *in vitro* study showed PPARδ overexpression or activation triggered neuronal stem cells to differentiate into neurons rather than glial cells. However, PPARδ’s effects on cell proliferation might also include glial cells, which have been shown to have an effect on depressive-like behavior (Hashioka et al., 2013). The inconsistent findings of cellular differentiation between *in vivo* and *in vitro* investigations might be attributed to some differences in the extracellular microenvironment, and some questions remain to be further investigated. As we didn’t observe whether PPARδ overexpression or activation still shows antidepressant effects in mice lacking adult neurogenesis by irradiation or genetic manipulation, it could not be determined that neurogenesis is necessary for the antidepressant effect of PPARδ in the present study.

Overall, the results of the present study indicate that upregulating hippocampal PPARδ by genetic transfection or a small molecule agonist displays an antidepressive effect and enhances hippocampal neurogenesis in the context of stress, which provides new insight into the neurobiology of depression. PPARδ could be a novel and promising target for developing new drugs for the treatment of depressive disorders, although more studies are needed to confirm its role of regulation in the depressive behaviors.

## Supplementary Material

For supplementary material accompanying this paper, visit http://www.ijnp.oxfordjournals.org/

## Statement of Interest

All of the authors do not have financial interests to disclose.

## Supplementary Material

Supplementary Figure S1A
