# Supplementary material for: Hippocampal PPARδ Overexpression or Activation Represses Stress-Induced Depressive Behaviors and Enhances Neurogenesis
Source: Int J Neuropsychopharmacol. 2015 Sep 10;19(1):pyv083. doi: 10.1093/ijnp/pyv083 (PMC4772271; doi:10.1093/ijnp/pyv083)
Supplement: Supplementary Figure S1A [file Supplementary_Information.doc]

**Supplementary Information**

**Supplementary Methods**

**Lentivirus generation**

Mouse PPARδ cDNA was RT-PCR amplified. The following primers were used: forward: 5’- GAGGATCCCCGGGTACCGGTCGCCACCATGGAACAGCCACAGGAGGAG -3'; reverse: 5’- TCCTTGTAGTCCATACCGTACATGTCCTTGTAGATTTCCTGG -3’ (1364 bp, Genbank: NM_011145). To produce GV287-mPPARδ-EGFP, the PCR fragments and the GV287-EGFP plasmid were digested with Age I and ligated with Age I. The plasmid was used to transform DH5α *Escherichia coli* for identification. For recovery of recombinant lentivirus-PPARδ-EGFP (LV-PPARδ-EGFP), using 100 μl of Lipofectamine 2000 mixed with 2.4 ml of opti-MEM, HEK293 cells were co-transfected with 20 μg of the GV287-EGFP plasmid with a cDNA encoding mPPARδ and 15 μg pHelper 1.0 and 10 μg pHelper 2.0 plasmid to generate the recombinant lentivirus, LV-PPARδ-EGFP. After 48 h, HEK293 cells were used to amplify virus, and the supernatant harvested from HEK293 cells was filtered at 0.45μm. The vrius amplification was repeated thrice. HEK293 cells were then transfected with serially diluted lentivirus and labeled HEK293 cells were counted to calculate the viral titer (2×10^9^ TU/ml) 7 days later.

**Behavioral tests**

Physical state index. The physical state index was measured as described previously (Alonso et al., 2004). Each mouse was evaluated weekly by experimenters unaware of the treatment group over the stress period. Each measure was scored by a scale from 1 to 3:a damaged state with piloerection and/or dirty fur was noted 1 and a health state was noted 3, while the intermediate state was noted 2.

Tail suspension test (TST)**.** The TST was conducted as described previously (Cryan et al., 2005). Briefly, mice were allowed to adapt to the testing room before testing. Each mouse was suspended by its tail with adhesive tape for 6 min in a soundproof box. The time of immobility was assessed by ANY-MAZE software. The immobility time during initial two minutes of the 6-min task was discounted, while the last 4-min was statistically analyzed.

Forced swimming test (FST). The FST was conducted as described previously (Zhou et al., 2011). Mice were individually placed into an open glass cylinder (28 cm diameter, 33 cm height) filled to a depth of 20 cm with water at 25±1°C. The immobility time during the last 4 min was scored by ANY-MAZE software. Immobility time was defined as each mouse remained floating with all limbs motionless, except for the ones necessary for breathing. Water was replaced between each trial.

Novelty-suppressed feeding (NSF). The NSF was carried out similar to a previous publication (Santarelli et al., 2003). TEach mouse was deprived of food for 24 h before test. The test apparatus consisted of a box (50×50×20 cm) with a fluorescent light (450 lux) placed at the center of the arena covered with about 2 cm of saw dust. A single food pellet (regular chow) was positioned on a white paper (11 cm in diameter) in the center of the box. Mice were individually placed in one corner of the box and the latency to feed was measured. The interest (chewing) was defined as the mouse biting the food using forepaws. Immediately after the test, each mouse was transferred to its home cage and the food consumed within 6 min was divided by its weight (home cage food consumption).

Open field test (OFT)**.** Mice were individually placed in one corner square of the open arena, which was constructed of a plastic plate (50cm length×50 cm wide×40cm deep) and divided into 144 squares. Illumination was made available through fluorescent lights at 450 lux, which was placed at the center of the arena. Each mouse was allowed to freely explore the open field. Line crossings (number of squares crossed with four paws) was counted during a 6 min period and distance traveled was recorded in a 5 min period. After each trial, the plate was cleaned with 70% EtOH.

Elevated plus maze (EPM). The EPM test was assessed based on a previously described methodology (Masood et al., 2008). Mice were individually placed at the center of the maze comprises 2 open arms and 2 enclosed arms across from each other (30 × 5 cm and 30 × 5 × 15 cm high, respectively), facing an enclosed arm. It was elevated 40 cm above the floor. Each mouse was allowed 1 min to roam on all the arms. Then the number of entries (with all four paws) and time spent in both open and enclosed arms were analyzed for 5 min.

**Cell culture**

Adult neural stem cells（NSCs）from the DG of 8 to 9-week-old female mice were dissected and cultured as reported (Babu et al., 2007; Guo et al., 2012). Adult NSCs of the 2nd and 10th passage were used for experiments. For infection of NSCs, LV-PPARδ-EGFP or LV-EGFP was transfected into cultured NSCs by adding at 5 μl/dish (diameter 3.5 cm). 24 h later, a fresh medium was replaced without lentivirus. After a continuous transfection with the lentivirus for 5 days, the number of EGFP^+^ cells was observed in 20X high-power fields under an immunoflurescence microscope. About 80% of NSCs with EGFP-positive at the MOI of 10 were used for subsequent experiments.

**Immunoblot and RT-PCR assay**

Dentate gyrus was dissected and verified as described previously (Zhang JC et al., 2014). Mice were sacrificed by cervical dislocation and brains were sliced in 1-mm-thick coronal sections. Bilateral tissue DG were punched out and stored at –80°C. Verification of DG dissection was done by analyzing TDO2 and DSP gene expression by RT-PCR. Protein/mRNA extractions and immunoblot/RT-PCR assay were conducted as previously described (Tang et al., 2014). For immunoblot, mouse DG were homogenized in 0.5 ml of RIPA buffer (50mM Tris-HCl (pH 7.4), 150 nM NaCl, 1mM EDTA, 1mM PMSF, along with 1% Triton X-100, 1% sodium deoxycholate, 0.1% SDS). After centrifugation at 12000 g for 15 min, the total protein was collected from the supernatant. The concentration of protein was determined by Coomassie blue-based assay and then the expression of PPARδprotein was assessed. Samples were separated in SDS-polyacrylamide gel electrophoresis and transferred onto a PVDF membrane. Membranes were blocked in blocking buffer and then incubated overnight at 4 °C with respective primary antibodies for rabbit anti-PPARδ（1:150, Santa Cruz）and anti-β-actin (inner control, 1:2000, Bioworld). After washing 3 times with TBST, each membrane was incubated for 2h with a secondary antibody (1:5000) at room temperature. The detection and quantification of specific bands were performed with enhanced chemiluminescence detection reagents. For RT-PCR assay, total RNA was extracted from the DG using Trizol reagents according to the operation manual. For synthesising cDNA, the mixture contained aliquots of total RNA (2μg), 0.2μg random hexamer primer, 1mM dNTP, 20U Rnasin, 200 UM-MuLV reverse transcriptase in 20μl of the reverse reaction buffer was incubated at 25 °C for 10 min, 42 °C for 60 min and then at 72 °C for 10 min to deactivate the reverse transcriptase. PCR was performed on an Eppendorf Master Cycler (Eppendorf, Germany). 1 μl RT-cDNA template was dissolved in 20μl reaction mixture containing 1×PCR buffer, 200μM dNTP, 20 pmol of each primer, 1.5mM MgCl_2_ and 0.5 U Taq DNA ploymerase. PCR conditions were as below: 94 °C for 1 min, followed by 30 cycles of 94 °C for 30 s, 55 °C for 30 s, and 72 °C for 1 min, with a final extension step of 72 °C for 7 min. The primers for PPARδ and β-actin were as follows: mouse PPARδ forward 5’- GTCATGGAACAGCCACAGGAGGAGACCCCT-3’ and reverse 5’- GGGAGGAATTCTGGGAGAGGTCTGCACAGC-3’(130 bp, nucleotides 273-402 in NM_011145.3, GeneBank), β-actin forward 5’-TCTTGGGTATGGAATCCTGTG-3’ and reverse 5’-ATCTCCTTCTGCATCCTGTCA-3’ (154 bp, nucleotides 876–1029 in NM_007393.3, GeneBank). Samples were separated by 2% agarose gel electrophoresis containing ethidium bromide. Quantification was determined by optical density of the band with an image analysis system (Tanon Science & Technology Co. Ltd., China).

**Supplementary References**

Alonso R, Griebel G, Pavone G, Stemmelin J, Le Fur G, Soubrie P (2004) Blockade of CRF(1) or V(1b) receptors reverses stress-induced suppression of neurogenesis in a mouse model of depression. Mol Psychiatry 9: 278-286.

Babu H, Cheung G, Kettenmann H, Palmer TD, Kempermann G (2007) Enriched monolayer precursor cell cultures from microdissected adult mouse dentate gyrus yield functional granule cell-like neurons. PLoS One 2: e388.

Cryan JF, Mombereau C, Vassout A (2005) The tail suspension test as a model for assessing antidepressant activity: review of pharmacological and genetic studies in mice. Neurosci Biobehav Rev 29: 571-625.

Guo W, Patzlaff NE, Jobe EM, Zhao X (2012) Isolation of multipotent neural stem or progenitor cells from both the dentate gyrus and subventricular zone of a single adult mouse. Nat Protoc 7: 2005-2012.

[Masood A](http://www.ncbi.nlm.nih.gov/pubmed?term=Masood A%5BAuthor%5D&cauthor=true&cauthor_uid=18456873), [Nadeem A](http://www.ncbi.nlm.nih.gov/pubmed?term=Nadeem A%5BAuthor%5D&cauthor=true&cauthor_uid=18456873), [Mustafa SJ](http://www.ncbi.nlm.nih.gov/pubmed?term=Mustafa SJ%5BAuthor%5D&cauthor=true&cauthor_uid=18456873), [O'Donnell JM](http://www.ncbi.nlm.nih.gov/pubmed?term=O'Donnell JM%5BAuthor%5D&cauthor=true&cauthor_uid=18456873) (2008) Reversal of oxidative stress-induced anxiety by inhibition of phosphodiesterase-2 in mice. [J Pharmacol Exp Ther](http://www.ncbi.nlm.nih.gov/pubmed/18456873#%23) 326:369-379.

Santarelli L, Saxe M, Gross C, Surget A, Battaglia F, Dulawa S (2003) Requirement of hippocampal neurogenesis for the behavioral effects of antidepressants. Science 301: 805-809.

Tang SS, Ji MJ, Chen L, Hu M, Long Y, Li YQ, Miao MX, Li JC, Li N, Ji H, Chen XJ, Hong H(2014) Protective effect of pranlukast on Aβ_1-42_-induced cognitive deficits associated with downregulation of cysteinyl leukotriene receptor 1. Int J Neuropsychopharmacol 17:581-592.

Zhang JC, Wu J, Fujita Y, Yao W, Ren Q, Yang C, Li SX, Shirayama Y, Hashimoto K (2014) Antidepressant effects of TrkB ligands on depression-like behavior and dendritic changes in mice after inflammation. Int J Neuropsychopharmacol 18(4) pii: pyu077.

Zhou QG, Hu Y, Wu DL, Zhu LJ, Chen C, Jin X (2011) Hippocampal telomerase is involved in the modulation of depressive behaviors. J Neurosci 34:12258-12269.

**Supplementary Legends**

**Fig. S1** Hippocampal PPARδactivation decreases depressive behaviors. Shown are the immobility time in the TST (a), latency to feed and home cage consumption index in NSF test (b) in the mice exposed to CMS for 21 d, and then CMS for another 21 d, accompanied by intraperitoneally injected with GW0742 (5, 10 mg/kg) (n=8-10). Data are mean±SEM. *^*^p*<0.05, *^**^p* < 0.01, compared with CMS plus vehicle.

**Fig. S2** PPARδ activation enhances hippocampal neurogenesis. (a) Representative BrdU-positive cells in the DG from control or CMS mice with vehicle, 5 or 10 mg/kg GW0742, Scale bars = 50 μm, and (b) Total number of BrdU^+^ cells per DG from all groups (n=4). Data are means ± SEM. *^*^p<0.05*, *^**^p<0.01*, compared with CMS plus vehicle.

**Fig. S3** PPARδ activation promoted proliferation and differentiation of NSCs *in vitro*. (a) Representatives of BrdU^+^-labeled cells of the NSCs after treatment with GW0742, and (b) Statistical graph shows the number of BrdU^+^ cells in different groups (n= 6). (c, d) Immunocytochemistry for NeuN was used to assess neural differentiation in NSCs treated with GW0742, shown are statistical data of NeuN^+^-positive neurons in different groups (n= 6). Data are mean ± SEM. *^*^p < 0.05, ^**^p< 0.01,* compared with control. Scale bars = 50 μm.

**Fig. S1**

**
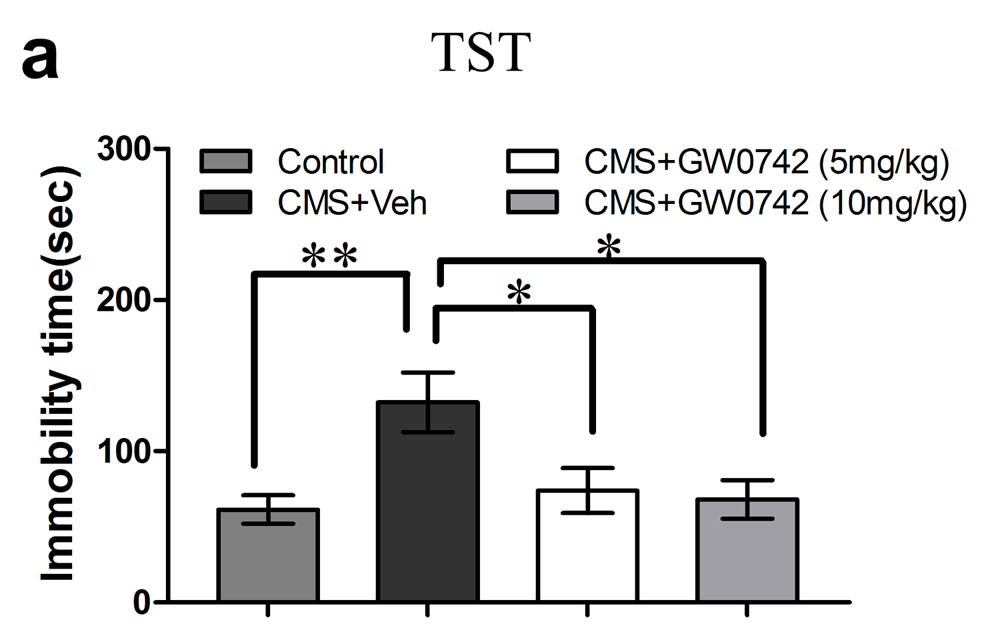
**

**
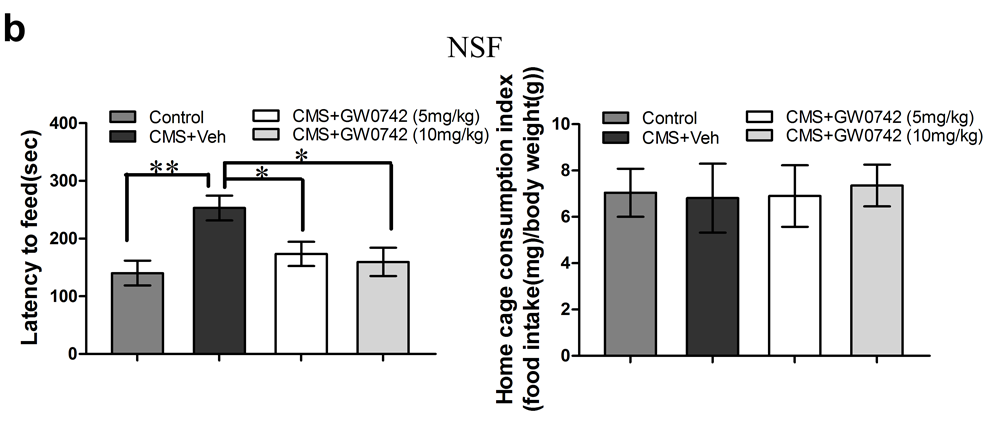
**

**Fig. S2**

**
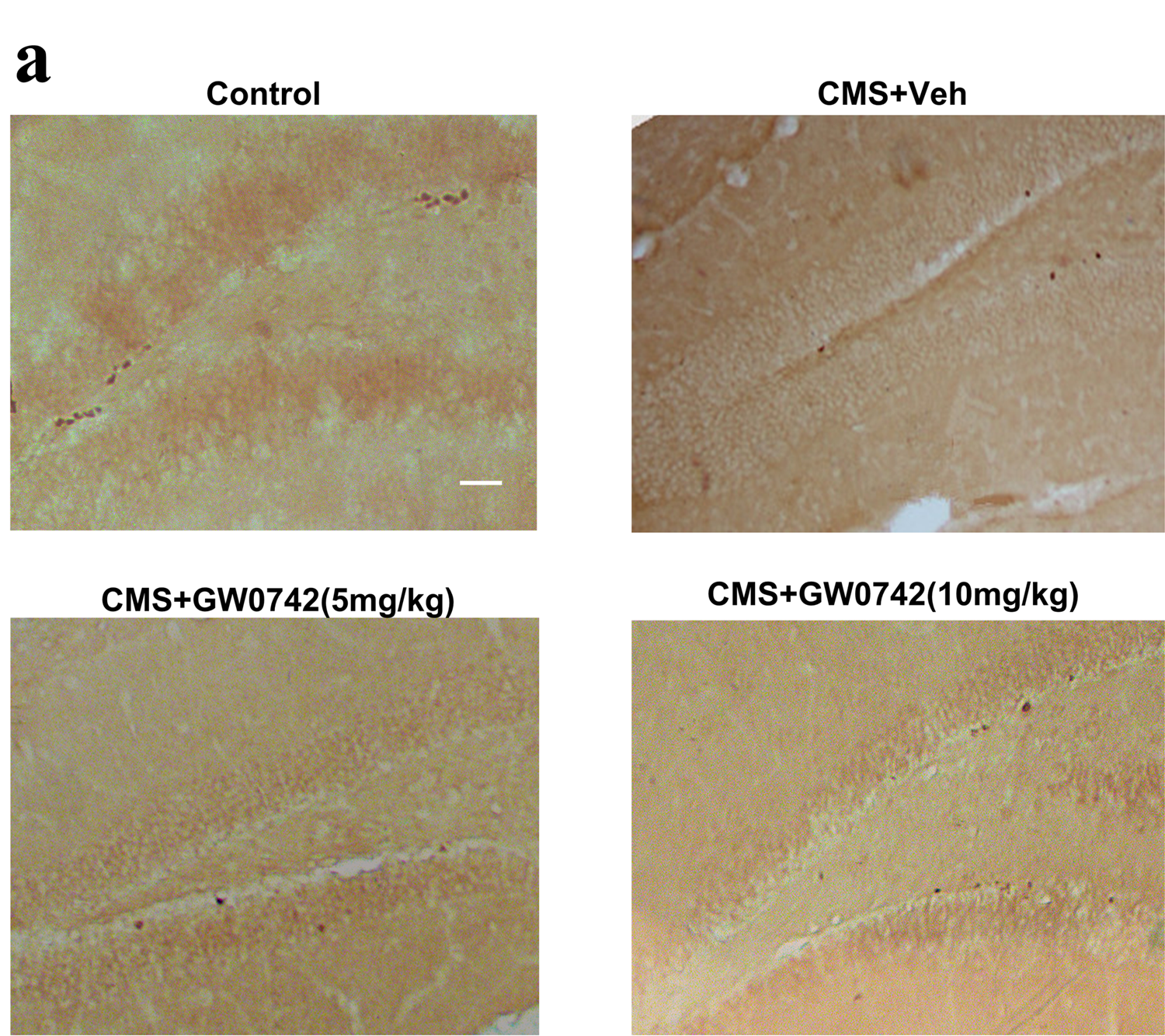
**

**
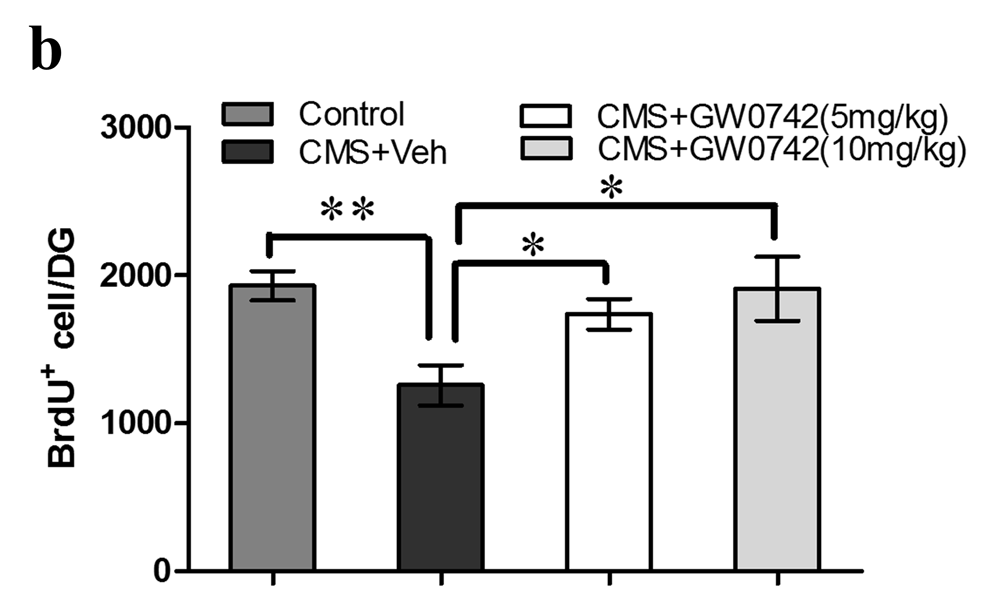
**

**Fig. S3**

**
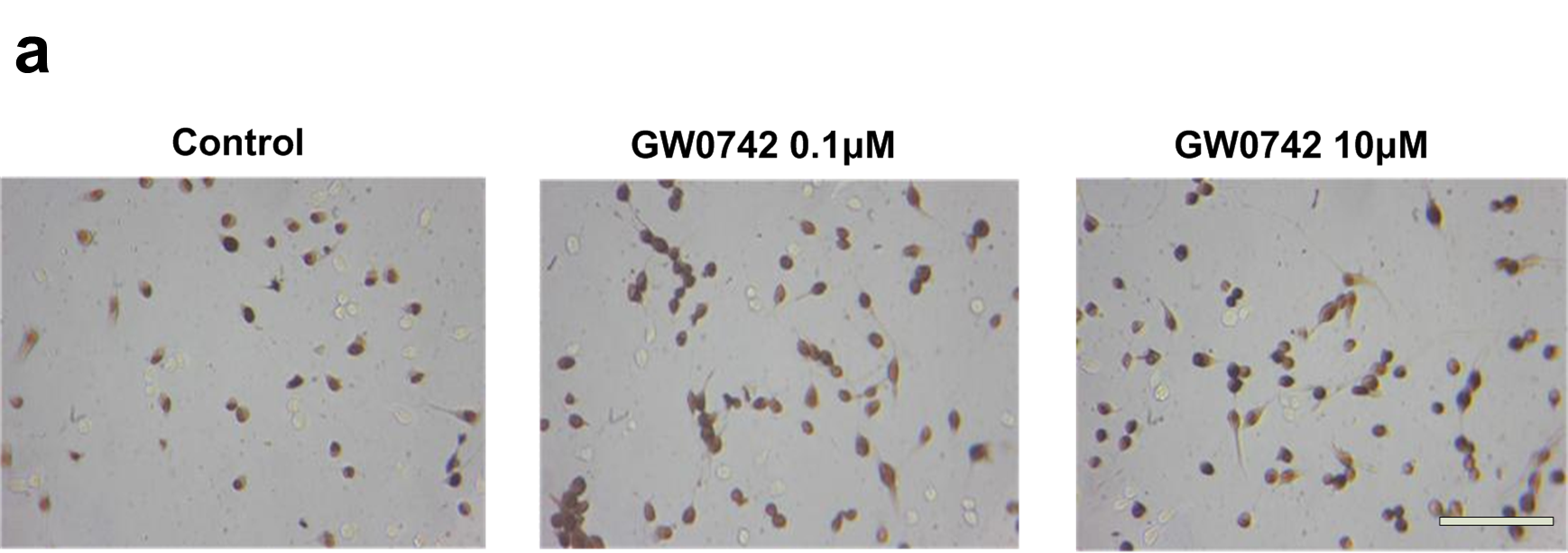
**

**
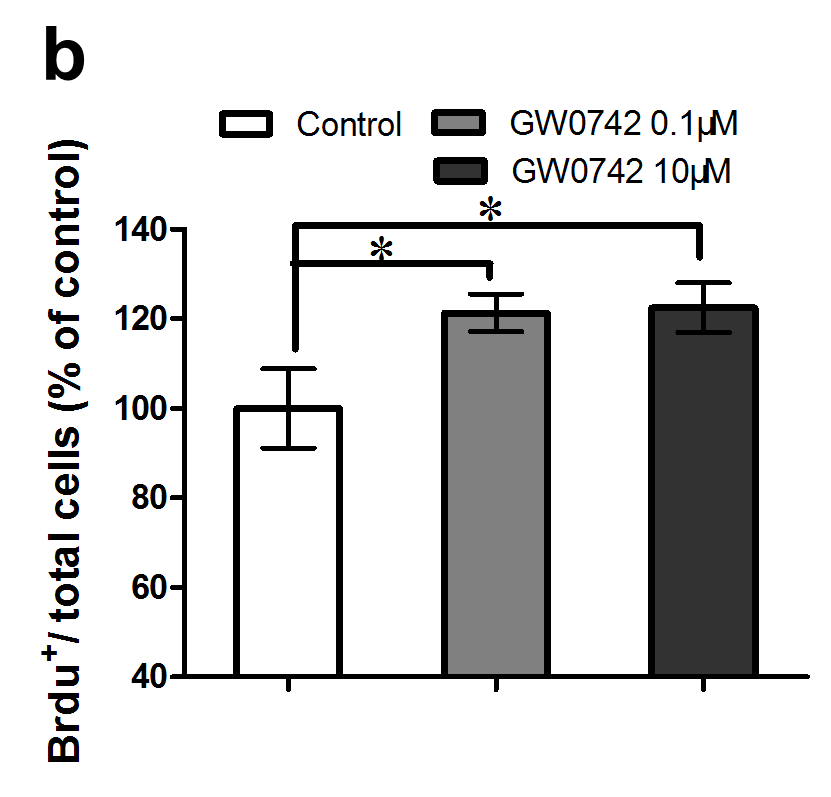
**

**
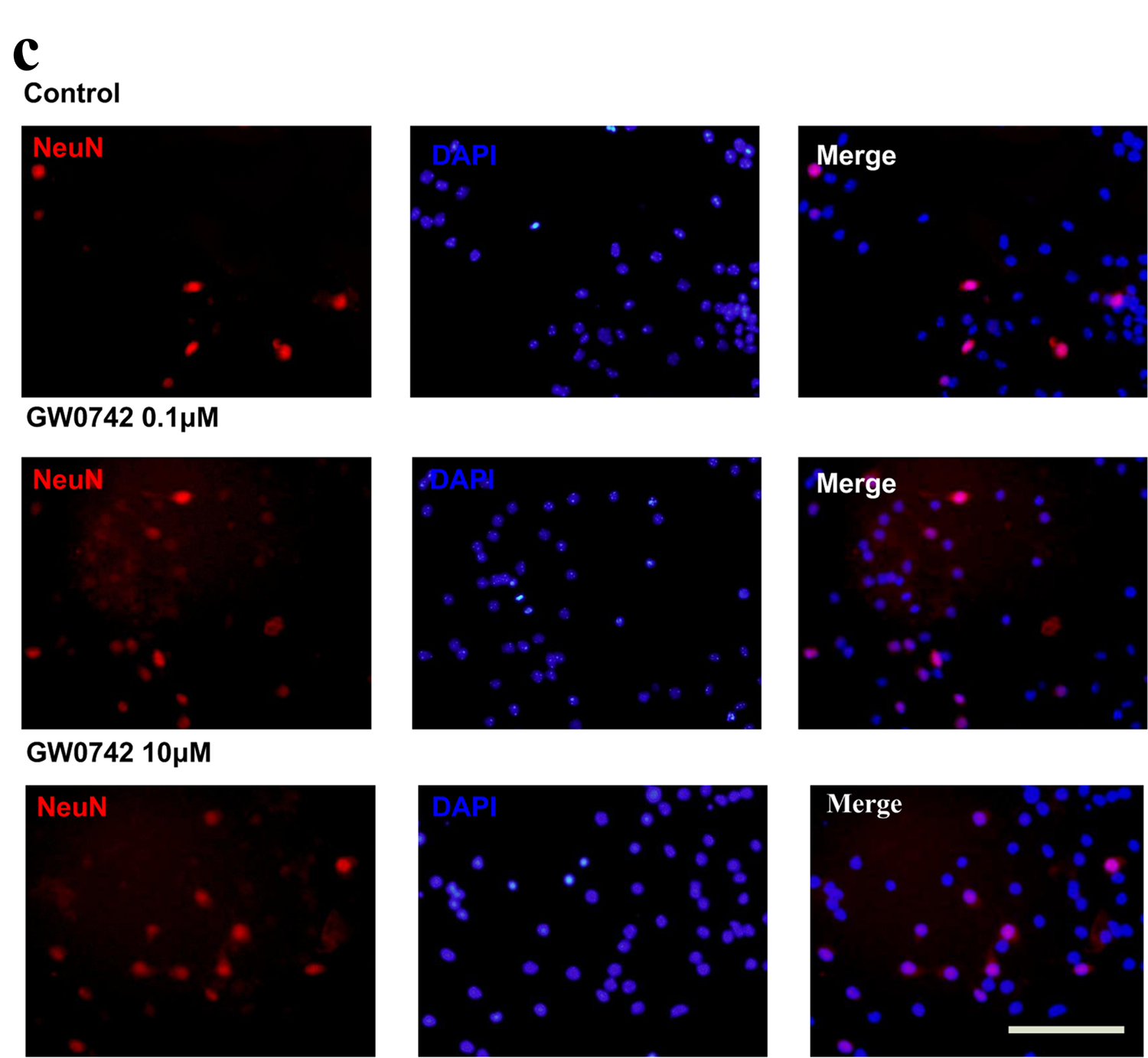
**

**
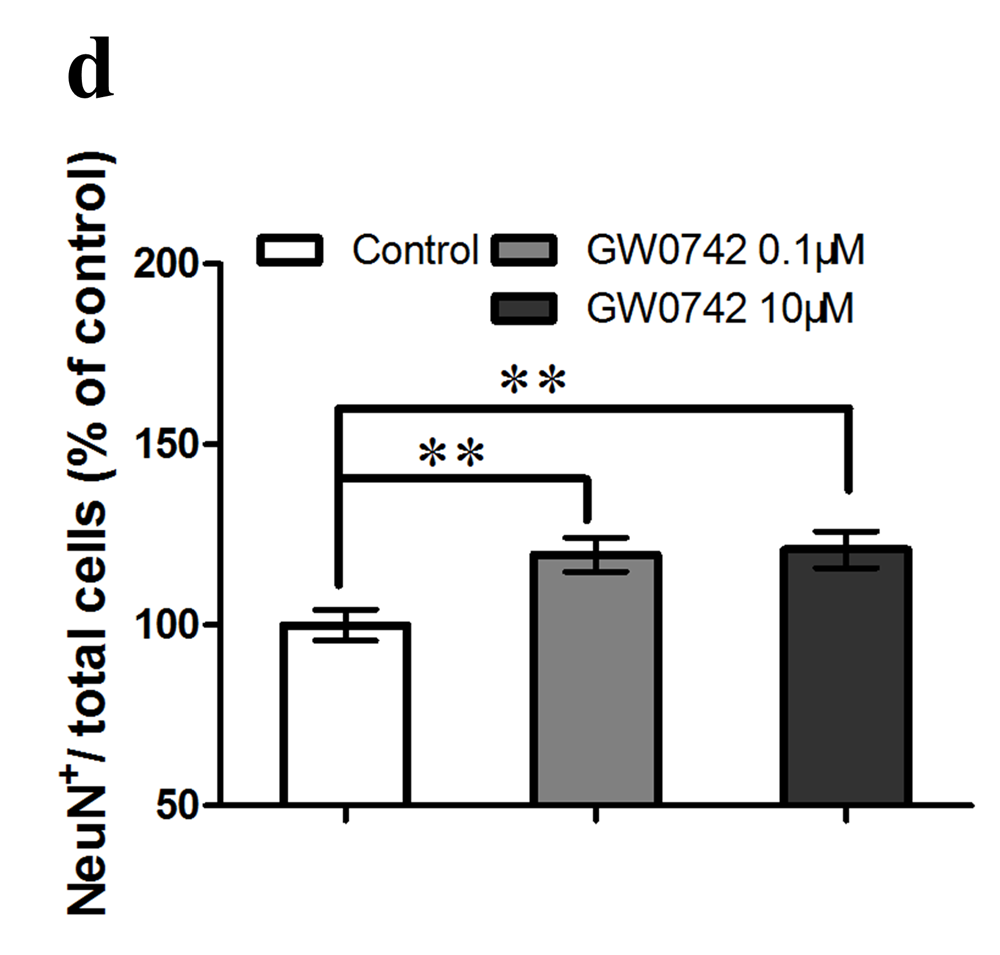
**
